# Cooperation or AI-driven self-reliance? Supplier channel exit decisions in live-streaming supply chain

**DOI:** 10.3389/frai.2026.1928289

**Published:** 2026-08-31

**Authors:** Wenyu Hou, Nan Chen

**Affiliations:** School of Management, Shanghai University, Shanghai, China

**Keywords:** AI streamers, dual-channel supply chain, game theory, live-streaming e-commerce, spillover effect

## Abstract

As AI technology rapidly matures, suppliers are actively establishing self-operated AI-hosted channels while continuing to rely on MCN resale channels. The cross-channel traffic spillover effect and consumers' AI preference have intensified channel conflicts, prompting some suppliers to consider exiting MCN partnerships. To examine the feasibility of such supplier exit behavior, this paper develops a two-stage Stackelberg game model between the supplier and the MCN. The analysis reveals that channel exit disrupts the inherent zero-sum competitive relationship within the supply chain, and that different combinations of spillover intensity and consumer AI preference yield differentiated equilibrium outcomes. Interestingly, when consumers exhibit strong preference for AI streamers, the MCN instead benefits from enhanced sales profits. Moreover, consumer channel preference tends to compress consumer surplus; however, a multi-party win can be achieved when a strong spillover effect coincides with low-to-moderate consumer AI preference. This study provides theoretical guidance for suppliers' channel-exit decisions and MCNs' response strategies, and offers practical references for livestream market regulation.

## 1 Introduction

Against the backdrop of deep integration between the digital economy and evolving consumption patterns, live-streaming e-commerce has emerged as one of the most dynamic sales channels in China's retail market. Data show that in 2025, the transaction volume of China's live-streaming e-commerce exceeded six trillion yuan, with user penetration surpassing 58%, profoundly reshaping the connection between brands and consumers.^1^ Unlike traditional e-commerce, which relies on static product images and text-based pages, live-streaming substantially shortens consumers' decision-making process through real-time interaction and scenario-based product demonstration (Bai et al., 2024). In this transformation, the rapid iteration of AI technology has injected new momentum into the live-streaming industry. Since the “618” shopping festival in 2023, major platforms including JD.com,^2^ Taobao,^3^ and Baidu Cloud^4^ have successively launched AI digital human live-streaming products. A Frost and Sullivan report indicates that the overall penetration rate of AI digital human live-streaming in China has exceeded 60%.^5^ Virtual digital human technology is progressively transitioning toward commercial deployment and is reshaping the traditional operational model of live-streaming supply chain.

As AI technology matures, an increasing number of suppliers are exploring self-operated AI live-streaming channels rather than relying exclusively on MCN agencies' resale channels for product distribution. This trend has given rise to a live-streaming supply chain model in which a self-operated AI channel and an MCN resale channel operate in parallel. In the beauty industry, for instance, brands such as Proya and Florasis engage top-tier MCN agencies like Li Jiaqi's studio and Bee Happy for major promotional campaigns, while simultaneously deploying proprietary AI digital human streamers in their official flagship store live-streaming rooms for routine sales, thereby achieving continuous consumer engagement during non-peak periods. In the early stage of dual-channel parallel operation, the supplier can leverage the MCN's traffic advantage to rapidly build market awareness, while simultaneously accumulating self-operational capabilities through the AI channel, generating a complementary synergy. However, this parallel structure can cause the consumer base cultivated in the MCN's live-streaming room to migrate toward the supplier's AI channel as brand awareness transfers, resulting in a live-streaming traffic spillover from the MCN channel to the supplier channel.

The existence of traffic spillover effects between dual live-streaming channels creates an intertwined competitive-cooperative channel relationship. On the one hand, a moderate spillover effect facilitates the growth of the supplier's AI channel and reduces market cultivation costs. On the other hand, when the spillover effect reaches a high level, the user base that the MCN has invested substantial operational resources in developing is appropriated by the supplier's channel, placing the MCN in a dilemma of cultivating demand that ultimately benefits a rival. For example, East Buy, while providing live-streaming sales services for multiple suppliers, accumulated a large base of brand followers and consumer data. However, suppliers such as Dongbei Nongsao subsequently launched their own branded live-streaming rooms on platforms such as TikTok and Rednote, deploying AI digital humans for routine operations and rapidly expanding the visibility of their self-operated channels by leveraging the traffic generated through East Buy. In 2026, Samsonite significantly scaled back its MCN collaborations and developed its own brand digital-human live-streaming system using JD JoyAI technology. China's rural e-commerce, constrained by high costs of MCN, has also turned to AI live-streaming with low entry barriers. For example, Zunyi tangerine sales, achieved solely through a self-built AI channel without MCN resellers, saw an 80% increase in sales efficiency. The resulting traffic redistribution has led some suppliers to reassess the long-term sustainability of dual-channel cooperation and to consider whether to terminate their partnership with the MCN in favor of a single-channel independent operation model centered on AI streamers.

As the live-streaming dual-channel model “MCN resale plus AI self-operation” matures over time, the supplier's dependence on the MCN's traffic advantage has diminished. Concurrently, with the rapid iteration of AI live-streaming technology, due to the advantages of full-time service coverage and standardized processes offered by the supplier, more consumers accept and favor AI streamers. Consequently, suppliers are becoming more proactive in operating their own AI channels to enhance brand awareness and channel control, thereby attracting consumers to purchase directly through self-operated channels. When consumer preference for AI streamers exceeds a certain threshold and the live-streaming traffic spillover effect from the MCN resale channel is sufficiently pronounced, the supplier has an incentive to terminate its cooperation with the MCN, remove the MCN resale channel from the live-streaming market, and retain only a single self-operated AI channel, thus gaining full control over user data and supply chain dominance. In summary, this paper aims to determine whether the supplier should disrupt the status quo of dual-channel competition and cooperation, then we analyze the pricing logic of the supplier and the MCN under channel-exit scenarios, and investigate how channel-exit behavior affects the cooperative relationship between both parties and social welfare. Accordingly, this study focuses on the following research questions:

- Should the supplier implement a channel-exit decision and terminate the partnership with the MCN?
- How does supplier's channel-exit behavior into live-streaming sales channels impact MCN operational management and consumer welfare?
- Can supplier's channel-exit behavior achieve a win-win outcome for multiple stakeholders?

To address these questions, we construct a two-stage live-streaming supply chain comprising a supplier, an MCN, and consumers, and conduct an in-depth examination of the supplier's strategic behavior regarding whether to exit the cooperation with the MCN in the second stage, enabling the AI-powered sales channel to monopolize the live-streaming market. First, incorporating the live-streaming traffic spillover effect and consumer preference for AI technology, we develop Stackelberg game models under two scenarios and obtain equilibrium solutions *via* backward induction. Second, we compare the profit preferences of the supplier and the MCN under different strategies and analyze the impact of key parameters on demand and profit. Finally, we examine the effect of the supplier's channel-exit behavior on pricing and consumer surplus, corroborate the theoretical results through numerical simulation, and derive managerial implications for firm decision-making.

This study makes three principal contributions. First, focusing on the live-streaming traffic spillover effect and consumer preference for AI technology, we construct a two-stage Stackelberg model for a supplier–MCN dual-channel supply chain. To the best of our knowledge, existing literature has predominantly concentrated on the collaborative relationship between KOLs and traditional retail firms and its short-term marketing effects. This study, grounded in the pivotal role of MCN agencies in the live-streaming system and incorporating the application of emerging technologies such as AI in firms' digital transformation, integrates the channel structure evolution mechanisms driven by consumer preferences for AI technology. This extends the research perspective in this field and further enriches the literature on dual-channel coordination and the integration of emerging digital technologies in the live-streaming e-commerce context. Second, this study revises several prevailing assumptions, dispels the MCN's adversarial stance toward competing channels driven by traffic spillover concerns, and advises suppliers to approach consumer preference rationally when determining cooperation modes. Third, this study reveals several novel and insightful findings. For example, we find the multi-dimensional impact of the supplier's channel-exit behavior on pricing, profit, and consumer surplus. Notably, under specific conditions, a reduction in the number of channels can paradoxically enhance consumer welfare. These findings deepen the understanding of welfare redistribution mechanisms in dual-channel competition.

## 2 Literature review

### 2.1 Live-streaming e-commerce

As an emerging real-time communication medium, live-streaming has integrated with various industries such as entertainment, education, and healthcare, while particularly revolutionizing conventional sales paradigms in the commercial sector (Mao et al., 2022). Consequently, it has attracted significant scholarly attention (Sun et al., 2019; Zhao and Fan, 2024). Research on live-streaming e-commerce primarily focuses on consumer purchase intention (Qu et al., 2023; Sun et al., 2024), product returns and after-sales service (Xu et al., 2026b), channel pricing strategies (Han et al., 2026; Lin et al., 2026), and regulatory measures for live-streaming (Wang and Tai, 2025). This section specifically reviews studies examining the factors influencing the live-streaming models into sales channels. Lu et al. (2025) find that manufacturer-KOL collaboration prompts retailer information sharing for live-streaming, generating spillover benefits, yet manufacturers must balance KOL influence with information sharing. Chen et al. (2025) explore the timing for online retailers to introduce live-streaming channels and find that live-streaming operation decisions depend entirely on the establishment cost of the new channel. Xu et al. (2026c) find manufacturers can boost profit by adopting highly intelligent digital human streamers to replace internal live staff at low costs. Li et al. (2025) discover that high levels of live-streaming efficiency incentivize brands to integrate live-streaming formats into traditional sales channels. Xu et al. (2026a) examine how retailers can employ AI live-streaming strategies to deter supplier market encroachment. The afore-mentioned literature primarily focuses on brands introducing collaborations with live streamers within a single channel or on dual-channel structures composed of traditional sales and live-streaming e-commerce channels. This paper specifically investigates suppliers' motivations for exiting live-streaming channels, constructs a multi-live-streaming-channel framework, and examines how spillover effects from reselling-channel live-streaming influence both suppliers' channel exit strategies and their direct self-operated live-streaming channels.

### 2.2 Dual-channel sales

Dual-channel sales strategy represents a prominent topic in supply chain management (Chiang et al., 2003; Xia et al., 2025), with numerous scholars examining how channel spillover effects influence dual-channel competition (Abhishek et al., 2016; Song et al., 2026). Yang et al. (2023) design scenarios where manufacturers and retailers respectively collaborate with KOLs, revealing that both positive and negative spillover effects from live-streaming commerce benefit manufacturers most when retailers partner with KOLs. Li et al. (2024) integrate Hotelling and signaling game models, demonstrating that while benefiting from positive spillover effects from competitors' live-streaming, online retailers still prefer independent live-streaming strategies despite the risk of conveying low-quality signals to consumers. Zhang et al. (2025) analyze cross-side and same-side spillover effects and platform KOL selection, finding that KOLs boost user engagement for small streamers despite high entry fees. Niu et al. (2023) examine the incentives generated by adopting AI live-streaming and KOLs in dual-channel collaboration. Strong network externalities yield superior profits for brands that utilize AI. Existing literature predominantly addresses the marketing activities of human live-streamers, as orchestrated by KOLs or MCNs. However, few studies examine the positive spillover effects arising from suppliers' shift from MCN-led to AI-driven live-streaming. This cross-entity traffic spillover not only transforms the zero-sum competition between suppliers and MCNs but also complicates the interactive relationship between the two live-streaming formats.

### 2.3 AI technology application

Driven by breakthroughs in generative artificial intelligence, large language models, and natural language processing, AI technologies are increasingly permeating the live-streaming e-commerce sector, fundamentally reshaping the productivity structure of live-streaming channels. These technologies enable round-the-clock, intelligent content generation and dynamic interaction within live-streaming rooms, serving as a core driver of the industry's digital and intelligent transformation (Chen and Zhang, 2025; Gao et al., 2023). As a salient front-end application of AI in live-streaming e-commerce, the deployment of virtual digital human streamers has sparked extensive academic discussion regarding consumer acceptance of and preference for AI-hosted live-streaming (Chen et al., 2026; Hu and Ma, 2023). Sun et al. (2024) examine how the degree of anthropomorphism in AI streamers' virtual appearance influences consumer purchase intention, demonstrating that lower perceived realism can undermine consumers' endorsement of the streamer's promotional behavior during purchase decisions. Given that AI has diversified the forms of online information, consumers are continually exposed to an information-rich environment. Xu and Ruan (2023) document that intense social pressure can enhance the appeal of AI broadcasters among their audience. Liu et al. (2025) focus on consumers' willingness to pay for live-streaming service offered by AI virtual streamers. The study reveals that distinct emotional states can affect consumers' preferences when choosing between online celebrities and AI streamers. Xiong et al. (2026) develop an analytical model revealing that AI streamers must achieve higher intelligence levels and operate for longer durations to surpass the profits generated by KOL-hosted live-streaming. Accordingly, AI is no longer regarded merely as an auxiliary technological tool; rather, it has emerged as a novel factor of production deeply embedded in the reconstruction of “people–goods–place” within live-streaming e-commerce, propelling the industry's evolution from experience-driven operations toward an intelligent ecosystem integrating algorithmic and data-driven mechanisms.

Building on consumer preference for and trust in emerging technologies such as AI, this paper investigates how consumer inclination toward AI channel shapes the supplier's strategic decisions, with the aim of examining whether a stable positive relationship exists between consumer preference and product purchase rate.

### 2.4 Gap knowledge

Previous research has yielded substantial progress in the areas of live-streaming commerce, dual-channel sales, and AI technology application. Existing studies mostly focus on static channel decisions, with limited capacity to characterize channel changes triggered by emerging technology shocks. We captures the multi-stage channel evolution mechanism empowered by AI technology and provides dynamic decision-making references for channel layout in live-streaming e-commerce. Table 1 summarizes the distinctions between the relevant literature and this study, highlighting our distinctive focus.

**Table 1:** The differences between relevant literature and this paper.

Literature	Live-streaming	Spillover effect	AI Streamer	Dual-channel	Two-stage pricing
Abhishek et al., 2016		√		√	
Liu et al., 2025	√	√	√		
Mao et al., 2022	√				√
(Niu et al. 2023)	√	√	√	√	
Xia et al., 2025	√			√	
Xiong et al., 2026	√	√	√		
Zhang et al., 2025	√	√			
Zhao and Fan, 2024	√	√			√
This paper	√	√	√	√	√

## 3 Model

**Figure 1:**
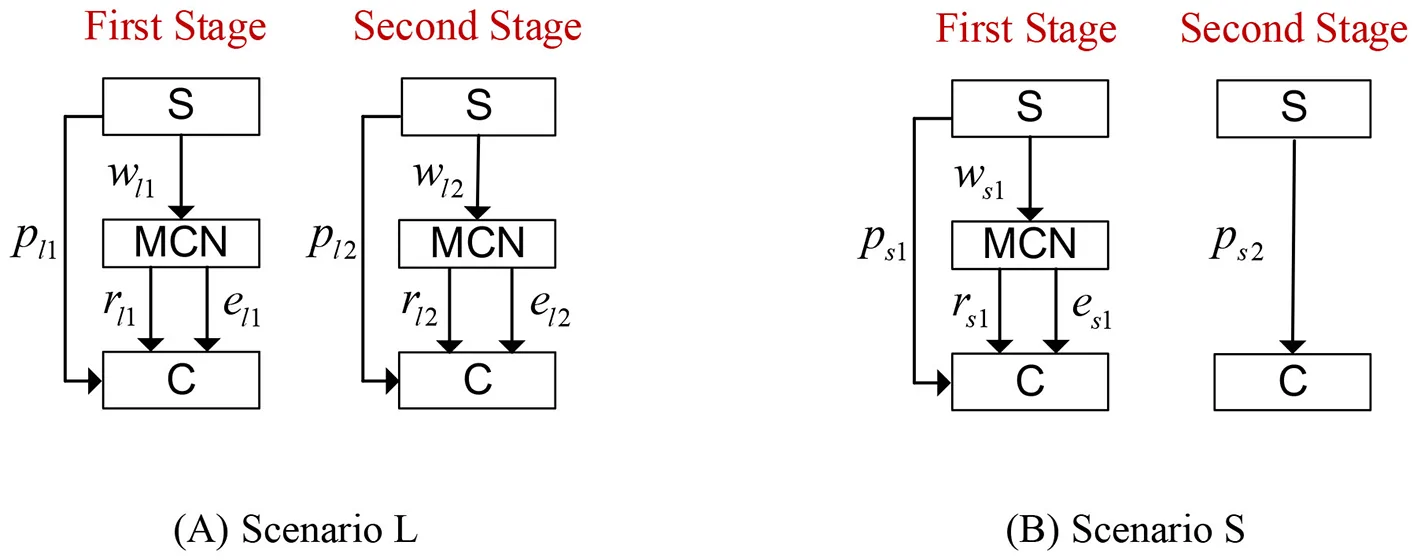
Supplier live-streaming strategies. **(A)** Long-term cooperation scenario. **(B)** Short-term cooperation scenario.

This paper considers a livestream supply chain system comprising a supplier, an MCN, and consumers. The supplier sells products to consumers through a self-operated AI live-streaming channel in both of two consecutive stages and must decide whether to cooperate with the MCN resale channel only in the first stage. Because MCN can generate a positive carryover effect from first-stage consumer demand to second-stage demand, we define the “inter-channel positive network effect that attracts additional consumers to purchase in the second stage” as the live-streaming traffic spillover effect. Benefiting from the first-stage traffic spillover, the supplier's brand awareness expands. While retaining the AI channel, the supplier may decide whether to terminate cooperation with the MCN and eliminate the MCN resale channel in the second stage. We define the “supplier's ability to terminate cooperation and revoke channel authorization at the end of the first stage” as the supplier's channel exit decision. The supplier holds the ultimate authorization right for the MCN resale channel. This implies that continued operation of the MCN resale channel hinges on the supplier's willingness to maintain authorization. If the supplier intends to terminate cooperation, the MCN cannot sustain the resale channel independently. The two resulting strategy combinations are illustrated in Figure 1.

### 3.1 Notation

To facilitate subsequent analysis, the notations employed in this study are defined in Table 2.

**Table 2:** Notations and their definitions.

Notation	Description
*w*_*ij*_	Wholesale price of the supplier
*p*_*ij*_	Retail price of the supplier
*r*_*ij*_	Retail price of the MCN
*e*_*ij*_	The MCN's live-streaming sales effort level
*v*	Customers' valuation of the product, $v^{∼}U(0,1)$
$U_{ij}^{h}$	Consumers' utility from buying the product
$q_{ij}^{h}$	Demand of the consumer
*CS*_*i*_	Consumer surplus at each strategy combination
*SW*_*i*_	Social welfare at each strategy combination
$\pi_{ij}^{h}$	The profit of member at each stage under different scenario
$\pi_{i}^{h}$	Total profit of each member
θ	The spillover effect of the MCN live-streaming, where 0 < θ < 1
*k*	Consumer preference for the supplier's AI streamers, where 0 < *k* < 1
*i*	Supplier's encroachment mode, where *i*∈{*l, s*}
*j*	Live-streaming sales stage, where*j*∈{1, 2}
*h*	Supply chain member, where *h*∈{*S, M*}

### 3.2 Model setup

In this study, consumers' perceived valuations of the product offered through the MCN channel and the supplier's AI-hosted channel are *v* and *kv*, respectively, uniformly distributed on the interval (0, 1), where*k*captures the degree of consumer preference for the AI streamer in the supplier's channel. Since AI streamers provide stable shopping services with round-the-clock interaction and convey more objective information than human streamers, consumers develop trust in the emerging technology and a preference for the service experience offered by AI live-streaming channels. In our model, a larger*k*indicates that consumers are more inclined toward the AI channel when choosing between the two channels. Another important parameter of our model is θ, which denotes the positive cross-channel traffic spillover effect generated by MCN live-streaming. This parameter characterizes the entire process of one-way traffic transfer from MCN live rooms in the first stage to the supplier's proprietary AI live rooms. The magnitude of θ reflects the scale of traffic transfer. A larger θ corresponds to more potential new customers flowing from the MCN channel to the AI channel, a higher click-through rate for the supplier's AI live room, and stronger brand exposure gains obtained by the supplier. $\theta q_{ij}^{M}$captures the demand increase enjoyed by the supplier in the current stage as a result of the traffic spillover from the preceding stage. Driven by the first-stage traffic acquisition, the supplier enters the livestream market and establishes a stable dual-channel sales arrangement with the MCN. Stimulated jointly by consumer preference for the AI channel and the traffic spillover, the supplier's channel grows steadily, prompting the supplier to consider terminating the partnership with the MCN, removing the MCN resale channel from the livestream market, and operating the self-owned AI-hosted channel as the sole interface with consumers. The MCN's sales effort *e* typically encompasses traffic promotion, consumer interaction experience, and after-sales service. The MCN's effort cost denoted by 1/2*e*^2^ (Li et al., 2025; Niu et al., 2023). To simplify the analysis and obtain clear managerial insights, we assume that the supplier's production cost is zero (Ji et al., 2023) and do not separately model the MCN commission rate. To ensure economically meaningful interior equilibrium solutions under both long-term cooperation and short-term cooperation strategies, we impose non-negativity constraints on all equilibrium demands for both channels and both stages ($q_{ij}^{h}>0$). Consequently, the spillover effect and consumer AI preference in this model satisfy θ_1_ < θ < θ_2_, The specific threshold values are shown in Appendix A.

The model consists of two consecutive selling stages, corresponding to two decision cycles of identical length. In both stages, the supplier sells products to consumers through the AI live-streaming channel while cooperating with the MCN resale channel in the first stage, forming a dual-channel competitive landscape. In real business scenarios, the first stage is often viewed as the supplier's traffic accumulation period, where the supplier leverages MCN live-streaming traffic to expand brand awareness. At this point, the utility a consumer derives from purchasing a product through the MCN channel is $U_{i1}^{M}=v-r_{i1}+\theta q_{i0}^{M}+e_{i1}$, and the utility from purchasing through the supplier's AI-hosted channel is $U_{i1}^{S}=kv-p_{i1}+\theta q_{i0}^{M}$, where $q_{i0}^{M}=1$ is assumed. Depending on whether cooperation continues in the second stage, two strategy combinations are considered. Under the long-term cooperation scenario, the supplier maintains the dual-channel structure with the MCN in the second stage, and the consumer's utilities from purchasing through the MCN channel and the supplier's channel are updated to $U_{l2}^{M}=v-r_{l2}+\theta q_{l1}^{M}+e_{l2}$ and $U_{l2}^{S}=kv-p_{l2}+\theta q_{l1}^{M}$, respectively. Under the short-term cooperation scenario, the supplier terminates the partnership in the second stage, the MCN channel exits the market, and the supplier retains only the self-operated AI-hosted channel, so that consumers face a monopoly market and the utility from product purchase becomes $U_{s2}^{S}=kv-p_{s2}+\theta q_{s1}^{M}$. In real business scenarios, the second stage is often viewed as the supplier's channel exit decision-making period, where the supplier needs to evaluate whether it is capable of independently serving the live-streaming market. Based on the utility functions, the dual-channel demand under the long-term and short-term cooperation scenarios is presented in Table 3.

**Table 3:** The demand functions under different scenarios.

Scenario	Demand function of the supplier	Demand function of the MCN
Scenario L	$q_{l1}^{S}=\frac{r_{l1}-p_{l1}-e_{l1}}{1-k}-\frac{p_{l1}-\theta q_{l0}^{M}}{k}$	$q_{l1}^{M}=1-\frac{r_{l1}-p_{l1}-e_{l1}}{1-k}$
	$q_{l2}^{S}=\frac{r_{l2}-p_{l2}-e_{l2}}{1-k}-\frac{p_{l2}-\theta q_{l1}^{M}}{k}$	$q_{l2}^{M}=1-\frac{r_{l2}-p_{l2}-e_{l2}}{1-k}$
Scenario S	$q_{s1}^{S}=\frac{r_{s1}-p_{s1}-e_{s1}}{1-k}-\frac{p_{s1}-\theta q_{s0}^{M}}{k}$	$q_{s1}^{M}=1-\frac{r_{s1}-p_{s1}-e_{s1}}{1-k}$
	$q_{s2}^{S}=1-\frac{p_{s2}-\theta q_{s1}^{M}}{k}$	—

The resale channel and the direct channel face the same market, and the market capacity is sufficiently large. Each consumer purchases at most one unit of the product in each stage, where 1 denotes the potential market demand size for the supplier's products (Chen et al., 2025). Under the long-term cooperation mode, because the dual-channel live-streaming structure remains stable, the two stages can be treated as a single integrated process. We assume that neither the supplier nor the MCN adjusts pricing or sales effort during the selling period. Based on the supply chain structures under the two cooperation strategies depicted in Figure 1, the profit functions of the supplier and the MCN are presented in Table 4.

**Table 4:** The profit functions under different scenarios.

Scenario	Profit function of the supplier	Profit function of the MCN
Scenario L	$\pi_{l}^{S}=w_{l1}q_{l1}^{M}+p_{l1}q_{l1}^{S}+w_{l2}q_{l2}^{M}+p_{l2}q_{l2}^{S}$	$\pi_{l}^{M}=\left(r_{l1}-w_{l1}\right)q_{l1}^{M}-\frac{1}{2}e_{l1}^{2}+\left(r_{l2}-w_{l2}\right)q_{l2}^{M}-\frac{1}{2}e_{l2}^{2}$
Scenario S	$\pi_{s}^{S}=w_{s1}q_{s1}^{M}+p_{s1}q_{s1}^{S}+p_{s2}q_{s2}^{S}$	$\pi_{s}^{M}=\left(r_{s1}-w_{s1}\right)q_{s1}^{M}-\frac{1}{2}e_{s1}^{2}$

### 3.3 Equilibrium analysis

**Figure 2:**
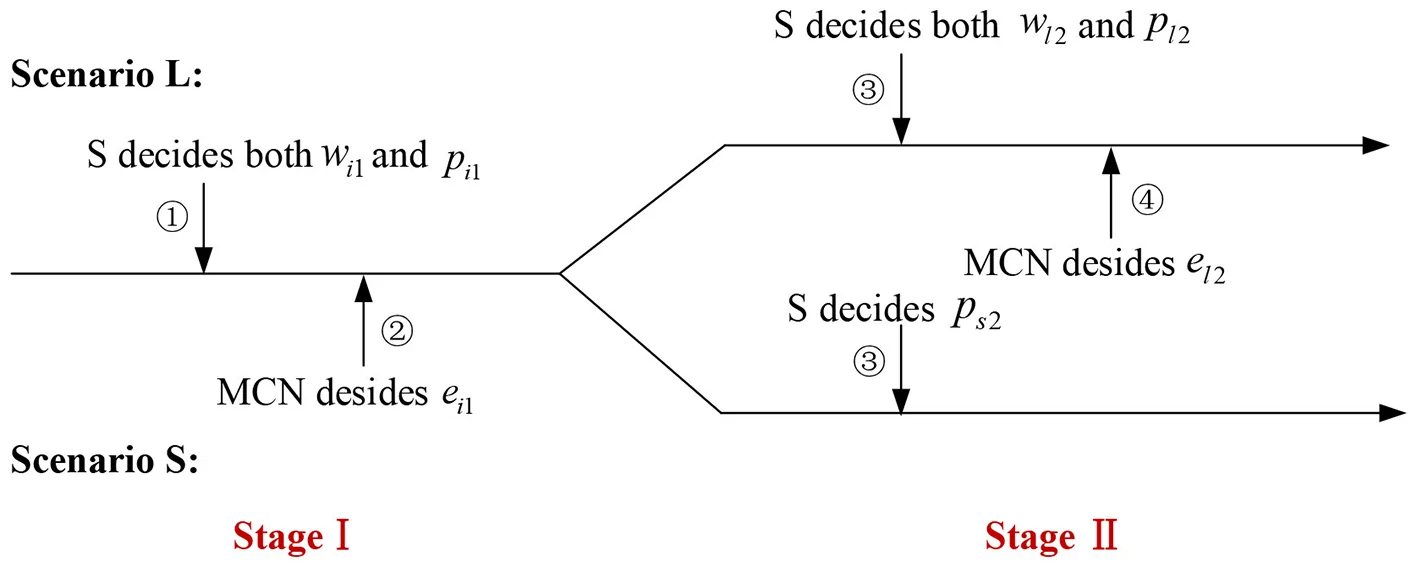
Sequence of events.

A Stackelberg game exists between the supplier and the MCN, where the supplier acts as the supply chain leader and the MCN acts as the follower. The sequence of events is illustrated in Figure 2. By maximizing the profits of the supplier and the MCN, the optimal dual-channel pricing strategies, livestream marketing effort, product demand, and firm profits under each cooperation mode can be determined. The detailed results are presented in Appendix A.

Building on the equilibrium solutions in Appendix A, we compare the strategic outcomes under the two channel exit strategies and examine how the long-term and short-term cooperation modes affect both parties' profits, yielding the following proposition.

*Proposition 1*: (1) When $\theta_{1}<\theta <{\bar{\theta }}_{1}$, both the supplier and the MCN prefer the long-term cooperation strategy. (2) When ${\bar{\theta }}_{1}<\theta <{\bar{\theta }}_{2}$, the supplier prefers long-term cooperation while the MCN prefers short-term cooperation. (3) When ${\bar{\theta }}_{2}<\theta <\theta_{2}$, both the supplier and the MCN prefer the short-term cooperation strategy.

Proposition 1 characterizes how the spillover effect governs the cooperation strategy preferences of the supplier and the MCN, revealing a shift from dual-channel to single-channel operation as the spillover intensifies. When the spillover effect is weak, the supplier's AI-hosted direct channel lacks sufficient market competitiveness on its own. Independent operation would expose the supplier to considerable trial-and-error costs and sales risks associated with channel entry, while the supplier still relies on the MCN's traffic spillover to sustain demand in its direct channel and cultivate consumer brand trust. For the MCN, the threat of demand diversion from the supplier's channel remains limited under weak spillovers. Maintaining the dual-channel structure enables the MCN to broaden its market reach, and a stable long-term partnership reduces its operational costs in supplier search and quality verification while signaling channel credibility to consumers.

As the spillover effect strengthens, the conversion rate of consumers from the MCN's live-stream room to the supplier's AI channel increases. With the supplier's direct channel growing increasingly mature, the MCN becomes less willing to continue subsidizing a competing channel. At the same time, the supplier's diminishing dependence erodes the MCN's wholesale bargaining power and raises its sales costs. To prevent the erosion of its market position, the MCN shifts its preference toward short-term cooperation, redirecting live-streaming traffic resources toward other supplier brands that have not yet established AI-hosted direct channels. When the spillover effect reaches a sufficiently high level, the supplier's AI channel has accumulated a stable consumer base and brand recognition through prior cooperation, satisfying the conditions for independent operation. With full control over pricing authority, the supplier avoids price competition with the MCN's channel and expands its profit margin. Consequently, the supplier also gravitates toward the short-term cooperation strategy as the spillover effect intensifies.

## 4 Sensitivity analysis

This section examines how consumer preference of AI technology and the spillover effect influence the wholesale price, the retail prices, MCN sales effort, market demand and profits. Several interesting corollaries can be derived from this analysis, while the corresponding proofs are provided in Appendix B.

### 4.1 Sensitivity analysis of consumer preference for the supplier's AI streamer

*Corollary 1*: (1) $\frac{\partial w_{l1}^{*}}{k}>0$; $\frac{\partial w_{s1}^{*}}{k}<0$. (2) $\frac{\partial p_{l1}^{*}}{k}>0$; $\frac{\partial p_{l2}^{*}}{k}>0$; $\frac{\partial r_{l1}^{*}}{k}>0$; $\frac{\partial r_{l2}^{*}}{k}>0$; $\frac{\partial p_{s1}^{*}}{k}>0$; $\frac{\partial p_{s2}^{*}}{k}>0$; $\frac{\partial r_{s1}^{*}}{k}>0$. (3) $\frac{\partial e_{l1}^{*}}{k}>0$; when $k_{1}<k<{\bar{k}}_{4}$, $\frac{\partial e_{s1}^{*}}{k}<0$, otherwise, $\frac{\partial e_{s1}^{*}}{k}>0$. (4) s$\frac{\partial q_{l1}^{M*}}{k}>0$; when $k_{1}<k<{\bar{k}}_{4}$, $\frac{\partial q_{s1}^{M*}}{k}<0$, otherwise, $\frac{\partial q_{s1}^{M*}}{k}>0$; $\frac{\partial q_{l1}^{S*}}{k}<0$; $\frac{\partial q_{l2}^{S*}}{k}<0$; $\frac{\partial q_{s1}^{S*}}{k}<0$; $\frac{\partial q_{s2}^{S*}}{k}<0$. (5) When $k_{1}<k<{\bar{k}}_{5}$, $\frac{\partial \pi_{l}^{S*}}{k}<0$, otherwise, $\frac{\partial \pi_{l}^{S*}}{k}>0$; when $k_{1}<k<{\bar{k}}_{6}$, $\frac{\partial \pi_{l}^{M*}}{k}<0$, otherwise, $\frac{\partial \pi_{l}^{M*}}{k}>0$; when $k_{1}<k<{\bar{k}}_{7}$, $\frac{\partial \pi_{s}^{S*}}{k}<0$, otherwise, $\frac{\partial \pi_{s}^{S*}}{k}>0$; when $k_{1}<k<{\bar{k}}_{8}$, $\frac{\partial \pi_{s}^{M*}}{k}<0$, otherwise, $\frac{\partial \pi_{s}^{M*}}{k}>0$.

Corollary 1 reveals that consumer preference for the supplier's AI-hosted channel does not unconditionally promote demand expansion and profit improvement in that channel; rather, it can create a window of opportunity for the MCN to gain an upper hand in channel competition. In most cases, a higher degree of AI preference motivates the MCN to increase its marketing effort. Under long-term cooperation, an intensified AI preference amplifies the competitive threat from the supplier's self-operated live-streaming channel, compelling the MCN to raise marketing effort as a defensive response. Under short-term cooperation, however, the MCN proactively reduces marketing effort to avoid driving traffic to a rival channel, given the uncertainty surrounding the continuation of the partnership. Only when consumer preference for the AI host exceeds a critical threshold does the supplier's wholesale price subsidy compensate for the MCN's market erosion loss, at which point the MCN is willing to increase effort to capture short-term gains.

Consequently, the demand variation in the MCN channel is closely tied to its marketing effort investment. By contrast, the supplier's channel demand consistently decreases as preference deepens, because the heightened preference triggers defensive competition from the MCN for the supplier's market share and simultaneously misleads the supplier into setting excessively high prices, thereby suppressing its own demand growth. Overall, the impact of preference on both parties' profits exhibits a non-monotonic pattern of first decreasing and then increasing. At low preference levels, intensified competition leads to demand distortion and pricing inefficiency: under long-term cooperation, the MCN's incremental marketing cost cannot be offset by the corresponding revenue gain, while under short-term cooperation, the MCN's passive live-streaming performance reduces sales volume and compresses the supplier's wholesale profit margin, causing profits to contract for both parties. When preference rises to a high level, the brand's aggregate premium pricing capacity strengthens, and the MCN's channel price and demand increase in tandem; the supplier likewise benefits from the elevated price, which offsets the declining sales volume, so that both parties achieve a Pareto improvement.

### 4.2 Sensitivity analysis of the live-streaming traffic spillover effect

*Corollary 2:* (1) When $\theta_{1}<\theta <{\bar{\theta }}_{3}$, $\frac{\partial w_{l1}^{*}}{\theta }>0$, otherwise, $\frac{\partial w_{l1}^{*}}{\theta }<0$; $\frac{\partial w_{s1}^{*}}{\theta }<0$. (2) $\frac{\partial p_{l1}^{*}}{\theta }>0$; $\frac{\partial p_{l2}^{*}}{\theta }>0$; $\frac{\partial r_{l1}^{*}}{\theta }>0$; $\frac{\partial r_{l2}^{*}}{\theta }>0$; $\frac{\partial p_{s1}^{*}}{\theta }>0$; $\frac{\partial p_{s2}^{*}}{\theta }>0$; $\frac{\partial r_{s1}^{*}}{\theta }>0$. (3) $\frac{\partial e_{l1}^{*}}{\theta }>0$; $\frac{\partial e_{s1}^{*}}{\theta }>0$. (4) $\frac{\partial q_{l1}^{M*}}{\theta }>0$; $\frac{\partial q_{s1}^{M*}}{\theta }>0$; $\frac{\partial q_{l1}^{S*}}{\theta }>0$; $\frac{\partial q_{l2}^{S*}}{\theta }>0$; $\frac{\partial q_{s2}^{S*}}{\theta }>0$; when $\theta_{1}<\theta <{\bar{\theta }}_{4}$, $\frac{\partial q_{s1}^{S*}}{\theta }>0$, otherwise, $\frac{\partial q_{s1}^{S*}}{k}<0$. (5) $\frac{\partial \pi_{l}^{S*}}{\theta }>0$,$\frac{\partial \pi_{s}^{S*}}{\theta }>0$, $\frac{\partial \pi_{l}^{M*}}{\theta }>0$, $\frac{\partial \pi_{s}^{M*}}{\theta }>0$.

Corollary 2 demonstrates that the live-streaming traffic spillover effect is conducive to the expansion and value enhancement of the dual-channel supply chain across the majority of dimensions, and does not erode the MCN's profit as a result of traffic leakage. An increase in the spillover effect implies that each additional unit of marketing investment by the MCN reaches a larger proportion of consumers in the supplier's channel, thereby motivating the MCN to intensify its marketing effort to stimulate sales and sustain the cooperative relationship with the supplier. Meanwhile, a growing spillover effect reflects the market potential of the supplier's product, prompting the MCN to deliver superior live-streaming performance in order to secure high-quality supply. Under long-term cooperation, each live-streaming session conducted by the MCN inadvertently provides free promotional exposure for the supplier's AI-hosted channel. As the spillover effect increases, the supplier gains greater bargaining leverage in the pricing game and raises the wholesale price; the MCN, in turn, tends to increase the retail price on the expanded demand base to pass through the higher procurement cost, resulting in a coordinated price increase by both parties. Even when the spillover effect becomes sufficiently high that the supplier concedes on the wholesale price to compensate the MCN for traffic loss, the retail price continues to rise alongside overall demand expansion. Under short-term cooperation, the supplier reduces the wholesale price to incentivize downstream sales expansion—thereby enlarging the profit base for the second-stage monopoly operation—while simultaneously raising the retail price to maintain stability across the two-stage pricing structure; the MCN increases its price to protect marginal profit.

Beyond the across-the-board price increase, the spillover effect also promotes demand expansion in both channels. The traffic that the MCN diverts to the supplier enhances brand awareness and, in reverse, reinforces consumer trust in the MCN's channel. The supplier's AI-hosted channel, in turn, benefits from the MCN's brand endorsement, which strengthens consumers' purchase intention. Under short-term cooperation, however, the supplier strategically constrains the first-stage expansion of the AI-hosted channel when the spillover effect is sufficiently high, so as to prepare for independent second-stage operation and demand absorption. The combination of demand expansion and higher retail prices drives a steady increase in the supplier's profit. The MCN, under long-term cooperation, collaborates with the supplier to shift away from low-price competition and implement a premium pricing strategy; under short-term cooperation, it benefits from the reduced wholesale price that lowers procurement costs. In both scenarios, demand growth and cost savings jointly ensure profit improvement.

## 5 Numerical simulation

To more intuitively illustrate how channel-exit behavior affects the wholesale and retail pricing decisions of the supplier and the MCN across different stages, as well as the impact of exit behavior on consumer welfare, this section specifies different values for *k* and θ, and analyzes the underlying drivers of the two-stage variations.

### 5.1 Impact of channel-exit behavior on pricing

**Figure 3:**
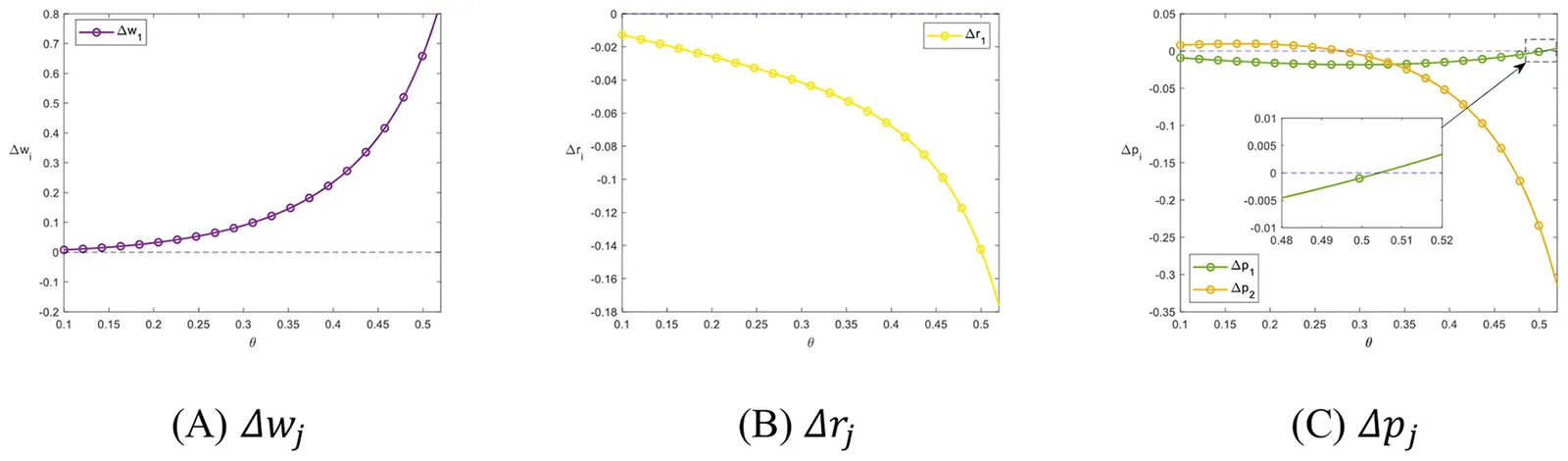
Impact of θ on the price difference. **(A)** Trend of supplier's wholesale price difference. **(B)** Trend of MCN's retail price difference. **(C)** Trend of supplier's retail price difference.

The strategic choice of any supply chain participant can drive cross-channel price fluctuations. To systematically examine the influence of the supplier's channel-exit behavior on wholesale and retail prices, we define Δ*w*_*j*_, Δ*r*_*j*_ and Δ*p*_*j*_ as the price gaps between long-term cooperation and short-term cooperation strategies within the same stage. To avoid relying merely on limited one-dimensional curves and achieve a comprehensive two-dimensional parameter analysis over the feasible (*k*, θ) domain, we first construct two-dimensional heat contour maps for each price difference across all valid parameter combinations, as summarized in Figure B1 of Appendix B. For intuitive illustration of typical price evolution patterns (Niu et al., 2023; Xiong et al., 2026), we further fix *k* = 0.15 and visualize how consumer preference for AI streamers affects two-stage pricing under the two strategic scenarios, as displayed in Figure 3.

*Proposition 2*: $w_{l1}^{*}>w_{s1}^{*}$. The supplier's wholesale price is consistently higher under the long-term cooperation strategy.

Proposition 2 indicates that the wholesale pricing discrepancy between the two strategies stems from differing strategic orientations toward market demand and firm profit. Under long-term cooperation, the supplier must secure wholesale profit in each stage to sustain the partnership, and the MCN is willing to accept higher wholesale costs in exchange for stable long-term live-streaming revenue. Under short-term cooperation, the supplier's second-stage monopoly profit depends on the MCN's first-stage sales volume—the higher the MCN's sales, the greater the number of potential users that spill over into the supplier's monopoly channel in the second stage. However, the channel-exit signal shortens the MCN's investment horizon, compelling the supplier to lower the wholesale price as a customer acquisition cost to incentivize the MCN's participation in the dual-channel partnership.

*Proposition 3*: $r_{l1}^{*}<r_{s1}^{*}$. The MCN's retail price is consistently lower under the long-term cooperation strategy.

Proposition 3 shows that the MCN's retail pricing under different strategies represents a differentiated mapping of the upstream wholesale price. Under long-term cooperation, despite the pressure of high procurement costs imposed by the supplier, the MCN forgoes passing these costs onto consumers in the first stage and instead lowers the retail price to lock in loyal followers and preemptively channel traffic into the second stage, thereby achieving profit growth over the entire cooperation period. Under short-term cooperation, the MCN lacks certainty regarding cross-period operations and tends to raise the retail price to maximize current-period revenue; the demand stimulus released by the supplier through a lower wholesale price therefore fails to reach consumers effectively.

*Proposition 4:* The impact of spillover effect intensity on the supplier's two-stage retail price under different cooperation strategies exhibits a significant stage-dependent reversal:

- First stage: When the spillover effect is weak, $p_{l1}^{*}<p_{s1}^{*}$; when the spillover effect is strong, $p_{l1}^{*}>p_{s1}^{*}$.
- Second stage: When the spillover effect is weak, $p_{l2}^{*}>p_{s2}^{*}$; when the spillover effect is strong, $p_{l2}^{*}<p_{s2}^{*}$.

Proposition 4 reveals that the spillover effect and cooperation strategy jointly shape the supplier's retail pricing across stages, but in opposite directions. Under weak spillover, in the first stage, the supplier under short-term cooperation raises AI channel price to divert consumers to the MCN and build a traffic pool, while the long-term cooperation lowers price to attract price-sensitive consumers. In the second stage, the supplier under short-term cooperation cuts price to stimulate monopoly demand, whereas the supplier under long-term cooperation maintains stable dual-channel prices. Under strong spillover, in the first stage, the supplier under long-term cooperation sustains a brand premium *via* shared traffic dividends, but the short-term cooperation faces MCN low-price pressure that forces price competition. In the second stage, the supplier under short-term cooperation shifts from low-price to quality premium using the first-stage traffic pool, while the long-term cooperation keeps prices lower due to channel rivalry.

Combining Propositions 2 through 4, the channel-exit decision complicates dual-channel competition and boosts information asymmetry, disturbing consumer price expectations and enabling dynamic pricing. Thus, wholesale and retail prices are dynamically governed by spillover effects, consumer AI preferences and cooperation expectations.

### 5.2 Impact of channel-exit behavior on consumer surplus and social welfare

Consumer surplus is defined as the difference between the maximum price a consumer is willing to pay for a unit of the supplier's product and its actual retail price (Li et al., 2024; Zhao and Fan, 2024). The formulas for consumer surplus under the two scenarios are presented as follows:


$$\begin{array}{l} CS_{l}=\int_{\frac{r_{l1}-p_{l1}-e_{l1}}{1-k}}^{1}\left(v-r_{l1}+\theta q_{l0}^{M}+e_{l1}\right)dv\\ \;+\;\int_{\frac{p_{l1}-\theta q_{l0}^{M}}{k}}^{\frac{r_{l1}-p_{l1}-e_{l1}}{1-k}}\left(kv-p_{l1}+\theta q_{l0}^{M}\right)dv\\ \;+\;\int_{\frac{r_{l2}-p_{l2}-e_{l2}}{1-k}}^{1}\left(v-r_{l2}+\theta q_{l1}^{M}+e_{l2}\right)dv\\ \;+\;\int_{\frac{p_{l2}-\theta q_{l1}^{M}}{k}}^{\frac{r_{l2}-p_{l2}-e_{l2}}{1-k}}\left(kv-p_{l2}+\theta q_{l1}^{M}\right)dv \end{array}$$ (1)

$$\begin{array}{l} CS_{s}=\int_{\frac{r_{s1}-p_{s1}-e_{s1}}{1-k}}^{1}\left(v-r_{s1}+\theta q_{s0}^{M}+e_{s1}\right)dv\\ \;+\;\int_{\frac{p_{s1}-\theta q_{s0}^{M}}{k}}^{\frac{r_{s1}-p_{s1}-e_{s1}}{1-k}}\left(kv-p_{s1}+\theta q_{s0}^{M}\right)dv\\ \;+\;\int_{\frac{p_{s2}-\theta q_{s1}^{M}}{k}}^{1}\left(kv-p_{s2}+\theta q_{s1}^{M}\right)dv \end{array}$$ (2)

**Figure 4:**
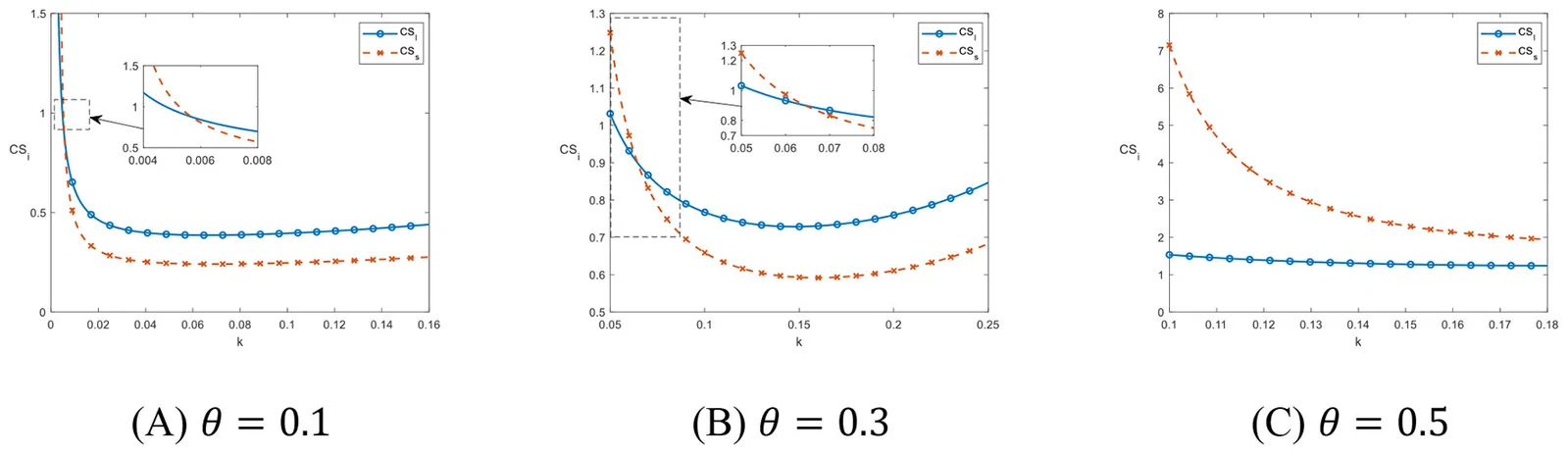
Impact of *k* on the consumer surplus.

For different intervals, we assign values to parameters: θ = 0.1; θ = 0.3; θ = 0.5. These three values span the full transition spectrum from consumers highly dependent on the MCN channel with significant cross-channel switching barriers to those with low MCN stickiness who migrate smoothly to the supplier's AI channel. Through numerical simulations, we examine the impact of consumer preference on consumer surplus, as shown in Figure 4.

From the consumer surplus perspective, rising AI channel preference generally depresses consumer surplus under both strategies, with a sharp initial drop followed by gradual stabilization. On the one hand, intensified inter-channel competition drives up aggregate retail prices, compressing consumer welfare. On the other hand, consumers' channel choices become increasingly entrenched: the long-term cooperation mode reinforces habitual viewing patterns and raises cross-channel switching costs, while the short-term cooperation mode narrows the range of available options by reducing the number of channels.

At low-to-moderate spillover, weak AI preference temporarily benefits surplus under short-term cooperation. But as preference grows, long-term cooperation uses stable dual-channel coordination to reduce price volatility and ultimately outperforms short-term cooperation. Thus, higher AI preference does not guarantee greater consumer surplus, which may instead trigger price hikes and conflict. The spillover effect moderates this tension. Consumers should avoid over-concentrating their preferences, as flexible choices foster competition and safeguard their welfare.

**Figure 5:**
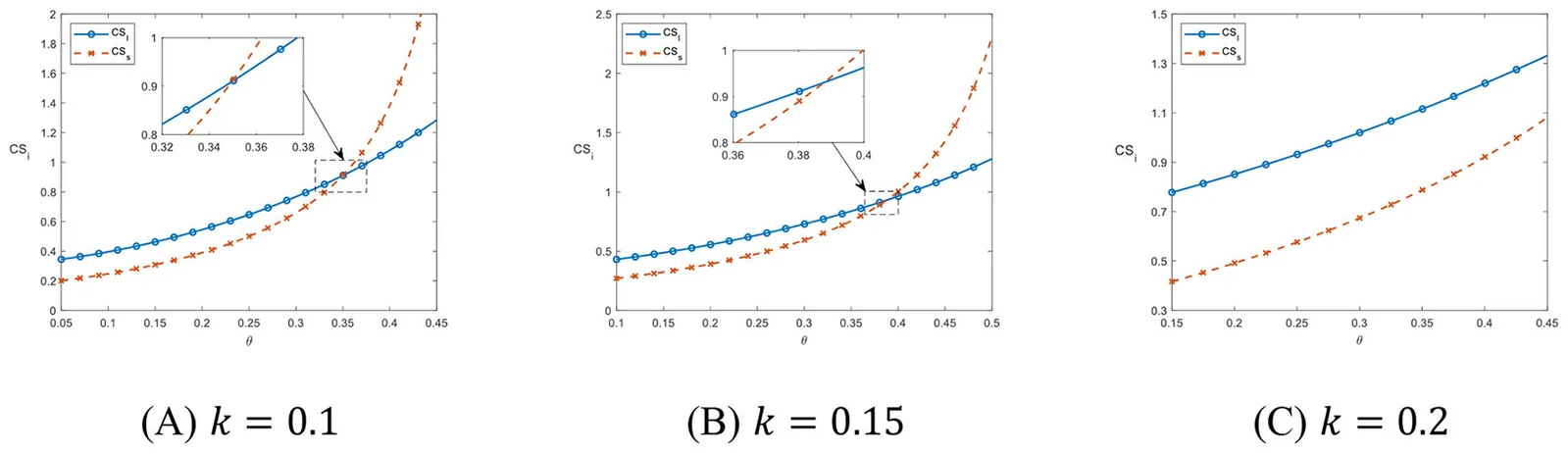
Impact of θ on the consumer surplus.

Similarly, to examine in detail the impact of the spillover effect on consumer surplus, we set specific parameter values as: *k* = 0.1, *k* = 0.18, *k* = 0.25. These three values represent a continuum of consumer preference, from human-streamer oriented to AI-streamer oriented. The results are presented in Figure 5.

The numerical simulation results indicate that, regardless of the level of consumer channel preference, consumer surplus under both strategies increases monotonically as the spillover effect strengthens. The spillover effect essentially expands product exposure and taps into potential consumers, lifting consumer surplus steadily.

Channel preference heterogeneity creates differentiated consumer surplus. At low and moderate AI channel preference, short-term cooperation yields higher consumer surplus once spillover exceeds a threshold. As spillover intensifies, live-streaming traffic offsets customer shortages. Free from binding cooperation, supplier gains far greater pricing flexibility and can offer more competitive prices than under long-term collaboration. In contrast, under strong channel preference, long-term cooperation consistently delivers superior consumer surplus, as it integrates the complementary strengths of MCN and AI streaming, stabilizes retail prices by averting channel-reduction volatility, and expands consumer choice.

Social welfare is defined as the sum of the total supply chain profit and consumer surplus, which comprehensively measures the overall economic welfare of the live-streaming dual-channel market (Zhao and Fan, 2024). The formulas for social welfare under the two scenarios are presented as follows:


$$\begin{array}{l} SW_{l}=CS_{l}+\pi_{l}^{S}+\pi_{l}^{M} \end{array}$$ (3)

$$\begin{array}{l} SW_{s}=CS_{s}+\pi_{s}^{S}+\pi_{s}^{M} \end{array}$$ (4)

*Proposition 5:* *SW*_*l*_<*SW*_*s*_. The social welfare gap widens significantly as the spillover effect intensifies, while narrowing gradually as consumer AI preference increases.

**Figure 6:**
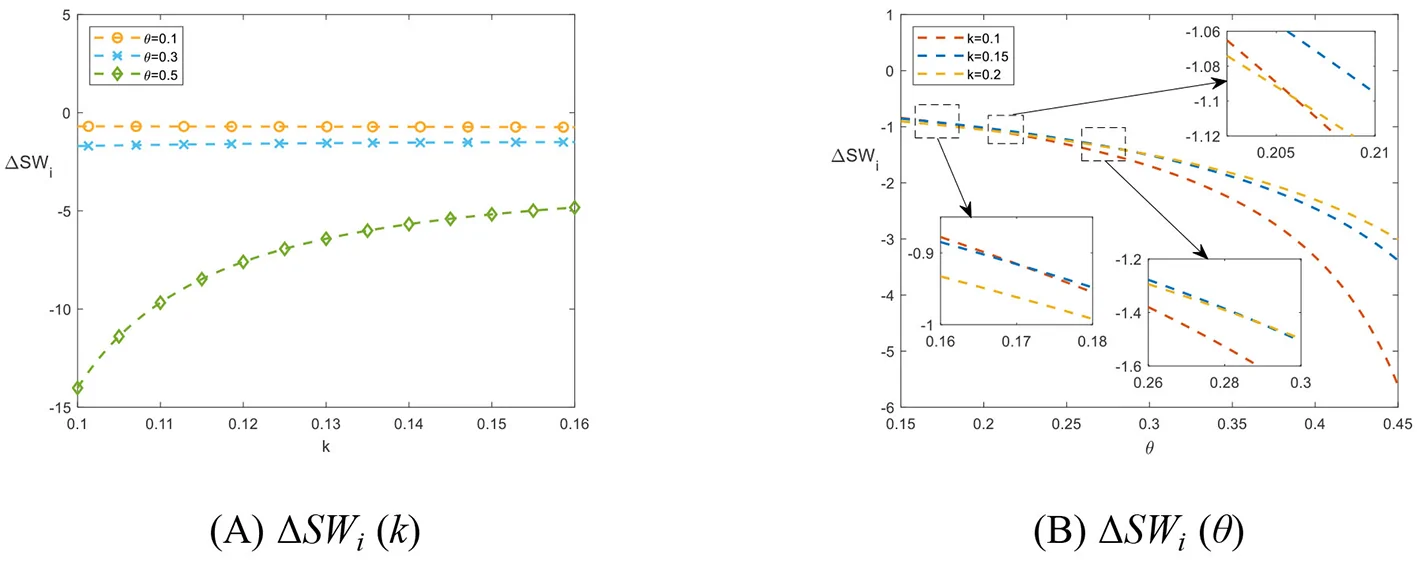
Impact of *k* and θ on the social welfare difference. **(A)** Impact of consumer AI preference on social welfare difference. **(B)** Impact of spillover effect on social welfare difference.

Overall, social welfare under short-term cooperation consistently exceeds that under long-term cooperation, while the spillover effect and consumer AI preference moderate the welfare gap between the two modes. An increase in the spillover effect allows the supplier in the short-term cooperation to independently set a competitive retail price based on AI channel consumer demand, thereby fully absorbing the spillover traffic and balancing firm profit with consumer surplus, which widens the social welfare gap. Notably, an increase in consumer AI preference plays an opposing moderating role. When consumers exhibit stronger preference for the AI live-streaming channel, the dual-channel coexistence structure under long-term cooperation better accommodates heterogeneous consumer demand. Human live-streaming operated by MCN and AI live-streaming provided by supplier form differentiated complements, covering a broader consumer base and narrowing the social welfare gap. The visualization results of Proposition 5 are presented in the Figure 6.

## 6 Conclusion

Incorporating factors such as the live-streaming traffic spillover effect and consumer preference for AI streamers, this paper develops a dual-channel supply chain model comprising a single supplier and a MCN, focusing on the channel-exit strategy after the dual-channel mode enters a stable development stage. Based on this framework, we analyze the impact of two cooperation modes on pricing, the MCN's live-streaming marketing effort, market demand, and dual-channel profits, and examine whether channel-exit behavior can achieve a multi-party win among the supplier, the MCN, and consumers. Through theoretical analysis, three main conclusions are drawn.

First, the spillover effect significantly influences the channel-exit strategy preferences of the supplier and the MCN. Contrary to the conventional belief that spillover necessarily erodes the source channel's profit, the spillover effect can simultaneously promote profit growth in both channels. When the spillover effect is weak, high trial and error costs and traffic anxiety lead both parties toward a fragile dual-channel cooperative equilibrium, forming a mutually dependent bargaining nexus. However, once the spillover effect increases substantially, single-channel independent operation becomes the jointly dominant strategy. The supplier, having accumulated a traffic pool through prior cooperation, gains the capability to operate independently and enhances its channel power. The MCN, to avoid subsidizing a rival channel and ceding its pricing leverage, also accepts short-term partnerships.

Second, the supplier's channel exit is not a zero-sum game. When consumer AI preference is at a low-to-moderate level, it can create a profit window for the MCN. This is because, to maximize the monopoly demand base in the second stage, the supplier must concede pricing power to the MCN at an extremely low wholesale price in the first stage in exchange for traffic accumulation and future channel dominance. The MCN thereby obtains a widening margin between procurement and retail prices, achieving a surge in short-term profit. From the perspective of the overall supply chain, channel exit eliminates the possibility of the MCN being progressively squeezed by a dominant AI channel in the future, enabling it to escape long-term competitive attrition and preserve strategic viability.

Third, under the combination of a high spillover effect and low-to-moderate consumer AI preference, the supplier's channel exit can achieve a multi-party win for upstream and downstream supply chain members and consumers. An extremely strong spillover effect fosters a negotiated consensus between both parties to reduce channels, resolving internal waste. Low-to-moderate AI preference means that consumers have not yet developed strong dependence on a single channel, which implies high migration elasticity. After independent operation, the supplier, compelled to compensate for its insufficient inherent attractiveness, passively lowers prices to capture market share, thereby releasing consumer welfare. However, excessively strong preference combined with forced channel reduction compels consumer migration, harming both the supply chain and consumer welfare.

By extracting insights from these findings, this paper offers theoretical contributions and practical references for suppliers executing live-streaming channel-exit decisions. The live-streaming traffic spillover effect does not necessarily harm the source channel's profit. For suppliers, before implementing channel exit decisions, they should systematically evaluate consumer acceptance of dual-channel and their own AI operational capabilities. For MCNs, they should enhance value-added services (e.g., creative content, emotional engagement) and diversify their client portfolios, thereby differentiating themselves in the multi-channel market and sustaining long-term cooperation with suppliers. For platforms, implement tiered governance: design traffic allocation balancing AI and human channels, and develop hybrid revenue-sharing contracts that align exit incentives with ecosystem health.

Although this paper yields several valuable findings, it comes with certain limitations. First, the model is built on a single-supplier and single-MCN bilateral framework, simplifying intricate multi-party competition in actual live-streaming market. Second, consumer surplus is analyzed primarily from the perspective of consumer preferences for emerging technologies. In reality, consumer utility is further influenced by live-streaming service quality, channel diversity, interactive experience and other factors.

Future research can introduce discount coefficients to optimize two-stage demand and profit under multi-supplier and multi-MCN competition for greater practical relevance. Besides, amid the booming AI live-streaming, investigating the impacts of government subsidies and platform governance rules on firms' channel strategies is a valuable research direction.

## Data Availability

The original contributions presented in the study are included in the article/Supplementary material, further inquiries can be directed to the corresponding author.

## Footnotes

1
http://sh.people.com.cn

2
https://www.jd.com

3
https://www.taobao.com

4
https://cloud.baidu.com

5
https://www.frostchina.com
