# Supplementary material for: Cooperation or AI-driven self-reliance? Supplier channel exit decisions in live-streaming supply chain
Source: Front Artif Intell. 2026 Aug 31;9:1928289. doi: 10.3389/frai.2026.1928289 (PMC13572583; doi:10.3389/frai.2026.1928289)
Supplement: Supplementary file 1 [file Data_Sheet_1.docx]

Supplementary Material

# 1 Appendix

Appendix A

The specific values of $\theta_{1}$ and $\theta_{2}$ are as follows.

|  | (1) |
| --- | --- |
|  | (2) |

The detailed results of equilibrium results are presented in Table A1.

Table A1 Optimal Solutions for Variables

| **Variables** | **First Stage** | **Second Stage** |
| --- | --- | --- |
| $w_{ij}$ | $w_{l1}^{*}=\frac{(6\theta+16)k^{2}-(3\theta^{2}+4\theta+8)k+2\theta^{2}}{32k^{2}-16k+\theta^{2}}$ | $w_{s1}^{*}=\frac{(4\theta+8)k^{2}-(\theta^{2}+2\theta+4)k+\theta^{3}+2\theta^{2}}{2(8k^{2}-4k+\theta^{2})}$ |
|  | $w_{l2}^{*}=\frac{(6\theta+16)k^{2}-(3\theta^{2}+4\theta+8)k+2\theta^{2}}{32k^{2}-16k+\theta^{2}}$ | — |
| $p_{ij}$ | $p_{l1}^{*}=\frac{16k^{3}+(10\theta-8)k^{2}-6k\theta}{32k^{2}-16k+\theta^{2}}$ | $p_{s1}^{*}=\frac{k+\theta}{2}$ |
|  | $p_{l2}^{*}=\frac{16k^{3}+(10\theta-8)k^{2}-6k\theta}{32k^{2}-16k+\theta^{2}}$ | $p_{s2}^{*}=\frac{4k^{3}+(\theta-2)k^{2}-k\theta}{8k^{2}-4k+\theta^{2}}$ |
| $r_{ij}$ | $r_{l1}^{*}=\frac{-8k^{3}+(8\theta+32)k^{2}-(2\theta^{2}+6\theta+16)k+\theta^{2}}{32k^{2}-16k+\theta^{2}}$ | $r_{s1}^{*}=\frac{-4k^{3}+(6\theta+16)k^{2}-(\theta^{2}+4\theta+8)k+\theta^{3}+2\theta^{2}}{2(8k^{2}-4k+\theta^{2})}$ |
|  | $r_{l2}^{*}=\frac{-8k^{3}+(8\theta+32)k^{2}-(2\theta^{2}+6\theta+16)k+\theta^{2}}{32k^{2}-16k+\theta^{2}}$ | — |
| $e_{ij}$ | $e_{l1}^{*}=\frac{8k^{2}-(2\theta+8)k-\theta^{2}}{32k^{2}-16k+\theta^{2}}$ | $e_{s1}^{*}=\frac{(2k-\theta-2)k}{8k^{2}-4k+\theta^{2}}$ |
|  | $e_{l2}^{*}=\frac{8k^{2}-(2\theta+8)k-\theta^{2}}{32k^{2}-16k+\theta^{2}}$ | — |
| $q_{ij}^{h}$ | $q_{l1}^{S*}=\frac{8k^{3}+24k^{2}\theta+(2\theta^{2}-10\theta)k+\theta^{3}}{\text{(32}k^{2}-16k+\theta^{2})k}$ | $q_{s1}^{S*}=\frac{4k^{3}+10k^{2}\theta+(\theta^{2}-4\theta)k+\theta^{3}}{2k(8k^{2}-4k+\theta^{2})}$ |
|  | $q_{l2}^{S*}=\frac{8k^{3}-2k\theta-\theta^{3}}{\text{(32}k^{2}-16k+\theta^{2})k}$ | $q_{s2}^{S*}=\frac{4k^{2}+(\theta-2)k-\theta}{8k^{2}-4k+\theta^{2}}$ |
|  | $q_{l1}^{M*}=\frac{8k^{2}-(2\theta+8)k-\theta^{2}}{32k^{2}-16k+\theta^{2}}$ | $q_{s1}^{M*}=\frac{(2k-\theta-2)k}{8k^{2}-4k+\theta^{2}}$ |
|  | $q_{l2}^{M*}=\frac{8k^{2}-(2\theta+8)k-\theta^{2}}{32k^{2}-16k+\theta^{2}}$ | — |
| $\pi_{i}^{h}$ | $\pi_{l}^{S*}=\frac{8k^{3}+(20\theta+8)k^{2}+(6\theta^{2}-12\theta-8)k-4\theta^{2}}{32k^{2}-16k+\theta^{2}}$ | $\pi_{s}^{S*}=\frac{20k^{3}\theta+(9\theta^{2}-12\theta-4)k^{2}+(2\theta^{3}-4\theta^{2})k+\theta^{4}+12k^{4}}{4k(8k^{2}-4k+\theta^{2})}$ |
|  | $\pi_{l}^{M*}=\frac{(1-2k)[8k^{2}-(2\theta+8)k-\theta^{2}]^{2}}{\text{(32}k^{2}-16k+\theta^{2})^{2}}$ | $\pi_{s}^{M*}=\frac{(1-2k)(2k-\theta-2)^{2}k^{2}}{2(8k^{2}-4k+\theta^{2})^{2}}$ |

We use backward induction to solve the model and obtain the equilibrium solutions. In the long-term cooperation model, we first take the first-order and second-order derivatives of the MCN's profit function$\pi_{l1}^{M}$ with respect to$e_{l1}$ and$r_{l1}$. Observing that$\frac{\partial^{2}\pi_{l1}^{M}}{\partial e_{l1}^{2}}=-2<0$ and$\frac{\partial^{2}\pi_{l1}^{M}}{\partial r_{l1}^{2}}=\frac{4}{k-1}<0$, it is straightforward to verify that an optimal solution exists. When the first-order conditions $\frac{\partial\pi_{l1}^{M}}{\partial e_{l1}}=\frac{r_{l1}-w_{l1}}{1-k}-e_{l1}=0$and$\frac{\partial\pi_{l1}^{M}}{\partial r_{l1}}=2-\frac{2(r_{l1}-w_{l1}-p_{l1}-e_{l1})}{1-k}=0$ hold, the MCN's optimal live-streaming marketing effort and retail price are derived as$e_{l1}^{*}=\frac{1-k+p_{l1}-w_{l1}}{1-2k}$ and$r_{l1}^{*}=\frac{(1-k)^{2}+(1-k)p_{l1}-kw_{l1}}{1-2k}$, respectively. Substituting $e_{l1}^{*}$ and $r_{l1}^{*}$ into the supplier's profit function $\pi_{l1}^{S}$ and taking the first-order and second-order derivatives with respect to $w_{l1}$ and $p_{l1}$, we observe that $\frac{\partial^{2}\pi_{l1}^{M}}{\partial w_{l1}^{2}}=\frac{4k}{2k^{2}-k}<0$ and $\frac{\partial^{2}\pi_{l1}^{M}}{\partial p_{l1}^{2}}=\frac{4-4k-2\theta}{2k^{2}-k}<0$, which confirm the existence of optimal solutions for the wholesale price and retail price under the supplier's AI channel. Substituting the resulting equilibrium solutions $w_{l1}^{*}$ and $p_{l1}^{*}$ back into $e_{l1}^{*}$ and $r_{l1}^{*}$ and solving, we then substitute the obtained equilibrium solutions into the demand functions and profit functions under the long-term cooperation mode to derive the corresponding market demands and optimal profits.

The solution process for the short-term cooperation model is the same as that for the long-term cooperation model. Therefore, we omit the details here.

The equilibrium solutions under the two strategies are summarized in Table A1.

Appendix B

Proof of Proposition 1

,.

When $\theta_{1}<\theta<\overline{\theta}_{1}$, then $\pi_{l}^{M*}>\pi_{s}^{M*}$; when $\overline{\theta}_{1}<\theta<\theta_{2}$, then $\pi_{l}^{M*}<\pi_{s}^{M*}$. When $\theta_{1}<\theta<\overline{\theta}_{2}$, then $\pi_{l}^{S*}>\pi_{s}^{S*}$; when $\overline{\theta}_{2}<\theta<\theta_{2}$, then $\pi_{l}^{S*}<\pi_{s}^{S*}$, where

 and.

Proof of Proposition 2

Based on the optimal equilibrium wholesale price of the supplier, and by performing difference calculations, we obtain:

.

Proof of Proposition 3

Based on the optimal equilibrium retail price of MCN, and by performing difference calculations, we obtain:

.

Proof of Proposition 4

Based on the optimal equilibrium retail price of the supplier, and by performing difference calculations, we obtain:

(1) When$\theta_{1}<\theta<\overline{\theta}_{5}$,, when$\overline{\theta}_{5}<\theta<\theta_{2}$,, where $\overline{\theta}_{5}=\frac{\sqrt{16k-47k^{2}}-k}{2}$.

(2) When$\theta_{1}<\theta<\overline{\theta}_{6}$,, when$\overline{\theta}_{6}<\theta<\theta_{2}$,, where $\overline{\theta}_{6}=\frac{3k-6k^{2}-\sqrt{k(1-2k)(198k^{2}-183k+40)}}{9k-5}$.

Figure B1 Two-dimensional heat contour maps on the price difference

|  |  |  |
| --- | --- | --- |
| (A) $\Delta w_{j}$ | (B) $\Delta r_{j}$ | (C) $\Delta p_{j}$ |

Proof of Corollary 1

By solving the first-order partial derivatives of the equilibrium solutions of the variables with respect to$k$, we obtain:

(1),.

(2),,,,,,.

(3), when$0<k<\frac{\theta\sqrt{3\theta^{2}+6\theta+4}-\theta^{2}}{4(\theta+1)}$, then, when$\frac{\theta\sqrt{3\theta^{2}+6\theta+4}-\theta^{2}}{4(\theta+1)}<k<1$, then.

(4); when$0<k<\frac{\theta\sqrt{3\theta^{2}+6\theta+4}-\theta^{2}}{4(\theta+1)}$, then，when$\frac{\theta\sqrt{3\theta^{2}+6\theta+4}-\theta^{2}}{4(\theta+1)}<k<1$, then ., ,, .

(5) ,,,.

When$0<k<\overline{k}_{1}$, then, when$\overline{k}_{1}<k<1$, then. When$0<k<\overline{k}_{2}$, then, when$\overline{k}_{2}<k<1$, . When$0<k<\overline{k}_{3}$, then, when$\overline{k}_{3}<k<1$, then. When$0<k<\overline{k}_{4}$, then, when$\overline{k}_{4}<k<1$, then.

, and

.

$\overline{k}_{6}=\frac{\sqrt{128u_{2}-30\theta^{2}-8\theta+16}+4-2\theta}{16}-\sqrt{\frac{(\sqrt{128u_{2}-30\theta^{2}-8\theta+16}+4-2\theta)^{2}}{256}-u_{2}-\frac{32(\theta-2)u_{2}+3\theta^{3}+60\theta^{2}}{32\sqrt{128u_{2}-30\theta^{2}-8\theta+16}}}$, and

.

$\overline{k}_{7}=Root\text{[}96Z^{6}-96Z^{5}+4(-9\theta^{2}+4\theta+8)Z^{4}+8(\theta^{3}+8\theta^{2})Z^{3}+(-15\theta^{4}-4\theta^{3}-20\theta^{2})Z^{2}+8\theta^{4}Z-\theta^{6}, i\text{]}$.

$\overline{k}_{8}=\frac{\sqrt{32u_{3}-6\theta^{2}-4\theta+4}+2-2\theta}{8}-\sqrt{\frac{(\sqrt{32u_{3}-6\theta^{2}-4\theta+4}+2-2\theta)^{2}}{64}-u_{3}-\frac{8(\theta-1)u_{3}+3\theta^{3}+10\theta^{2}}{8\sqrt{32u_{3}-6\theta^{2}-4\theta+4}}}$, and

.

Proof of Corollary 2

By solving the first-order partial derivatives of the equilibrium solutions of variables with respect to$\theta$, we obtain:

(1) When$\theta_{1}<\theta<\overline{\theta}_{3}$, then, when$\overline{\theta}_{3}<\theta<\theta_{2}$, then, where; .

(2), , , , , , .

(3) , .

(4) , . , . When$\theta_{1}<\theta<\overline{\theta}_{4}$, then, when$\overline{\theta}_{4}<\theta<\theta_{2}$, then; , where and .

(5),, , .
